# High water availability increases the negative impact of a native hemiparasite on its non-native host

**DOI:** 10.1093/jxb/erv548

**Published:** 2015-12-23

**Authors:** Robert M. Cirocco, José M. Facelli, Jennifer R. Watling

**Affiliations:** ^1^School of Biological Sciences, The University of Adelaide, SA 5005,Australia; ^2^Faculty of Health and Life Sciences, Northumbria University, Newcastle upon Tyne, Tyne and Wear NE1 8ST, UK

**Keywords:** Biomass, carbon isotope, nitrogen, parasitic plant–host interactions, photoinhibition, sodium, water availability.

## Abstract

Environmental factors alter the impacts of parasitic plants on their hosts. However, there have been no controlled studies on how water availability modulates stem hemiparasites’ effects on hosts. A glasshouse experiment was conducted to investigate the association between the Australian native stem hemiparasite *Cassytha pubescens* and the introduced host *Ulex europaeus* under high (HW) and low (LW) water supply. *Cassytha pubescens* had a significant, negative effect on the total biomass of *U. europaeus,* which was more severe in HW than LW. Regardless of watering treatment, infection significantly decreased shoot and root biomass, nodule biomass, nodule biomass per unit root biomass, *F*_v_/*F*_m_, and nitrogen concentration of *U. europaeus*. Host spine sodium concentration significantly increased in response to infection in LW but not HW conditions. Host water potential was significantly higher in HW than in LW, which may have allowed the parasite to maintain higher stomatal conductances in HW. In support of this, the δ^13^C of the parasite was significantly lower in HW than in LW (and significantly higher than the host). *C. pubescens* also had significantly higher *F*_v_/*F*_m_ and 66% higher biomass per unit host in the HW compared with the LW treatment. The data suggest that the enhanced performance of *C. pubescens* in HW resulted in higher parasite growth rates and thus a larger demand for resources from the host, leading to poorer host performance in HW compared with LW. *C. pubescens* should more negatively affect *U. europaeus* growth under wet conditions rather than under dry conditions in the field.

## Highlight

The Australian native stem hemiparasite, *Cassytha pubescens*, strongly reduced growth of the major introduced host *Ulex europaeus* in low water conditions and more severely so in high water conditions.

## Introduction

Parasitic plants are an important and diverse functional group that can have significant impacts on all ecosystems inhabited by higher plants. For example, mistletoes have been identified as keystone species in a number of habitats where they contribute to biodiversity by providing habitat and food sources for a range of organisms including birds, which, in turn, pollinate flowers and aid seed dispersal of both hosts and mistletoes (Watson, 2001; van Ommeren and Whitham, 2002; Mathiasen *et al.*, 2008). Parasitic plants can also influence nutrient cycling in the ecosystems where they occur (March and Watson, 2007; Mathiasen *et al.*, 2008). For instance, in the nutrient-poor soils of the sub-arctic, litter of the root hemiparasite *Bartsia alpina*, can create fertile patches that enhance the growth of surrounding vegetation (Quested *et al.*, 2003; Press and Phoenix, 2005). Parasitic plants may also function as viable bio-controls as native hemi- and holoparasitic vines in Australia and China, respectively, have been found to have a much greater negative impact on growth of introduced (non-native) plants, compared with native host species (Prider *et al.*, 2009; Li *et al.*, 2012).

Differential impacts of parasites on native and introduced hosts may be driven by how effectively parasites connect to and remove resources from their host’s vasculature via haustoria. The removal of host resources and subsequent effects on host performance are also influenced by a number of other factors including abiotic conditions. For instance, a high nitrogen supply has been found to dampen the effect of the stem holoparasite *Cuscuta reflexa* and the root hemiparasite *Striga hermonthica* on some hosts (Cechin and Press, 1993, 1994; Jeschke and Hilpert, 1997). While there are numerous studies on how nutrient supply affects the host–parasite relationship, there are surprisingly few studies investigating how water availability modulates the effects of the parasites on their hosts (Evans and Borowicz, 2013; Le *et al.*, 2015).

Using climate as a proxy for water availability, some studies have addressed water effects on associations involving mistletoes. In wetter environments, mistletoes tend not to maintain significantly higher transpiration rates or stomatal conductances than their hosts, which can affect their ability to withdraw resources from the host (Strong and Bannister, 2002). By contrast, in arid zones, mistletoes tend to have higher transpiration rates and stomatal conductances than their hosts, but they also track host transpiration (Ullmann *et al.*, 1985; Ehleringer *et al.*, 1986). Such co-ordination with the host may be necessary to prevent over-exploitation of water which would decrease the chances of survival for the host, and thus the parasite, in more arid conditions (Ullmann *et al.*, 1985; Miller *et al.*, 2003). However, despite this co-ordination, there may be some conditions that are just too harsh for parasites successfully to establish on hosts. In a study of mistletoes infecting *Eucalyptus largiflorens* in semi-arid southern Australia, Miller *et al.* (2003) found that rates of mistletoe infection were higher in less stressed hosts growing in more hydrated conditions. They suggested that increasing water stress made *E. largiflorens* a less suitable host for mistletoes. This also raises the question of whether parasite performance is improved when growing on more hydrated hosts and whether, as a result, the parasite has a greater effect on host performance in these conditions.

To our knowledge, there have been no experimental studies of how water influences the effects of stem hemiparasites on hosts, mainly because mistletoes typically infect trees which would be difficult to use in controlled experiments. This study used a stem hemiparasite that infects shrubs and thus is suitable for such experimental manipulations. The results of a glasshouse experiment are reported here for the effects of the Australian native stem hemiparasite *Cassytha pubescens* on the physiology and growth of the introduced host *Ulex europaeus* in high water (HW) and low water (LW) conditions (see Supplementary Figs S1 and S2 at *JXB* online). Parasite performance in both treatments was also measured. It was predicted that *C. pubescens* would have a negative effect on this host and that it would be more pronounced in HW compared with LW treatment due to a better parasite performance when water availability was high.

## Materials and methods

### Study species

*Ulex europaeus* L. (Fabaceae) is a perennial, evergreen, leguminous shrub that reaches 1–4 m in height (Clements *et al.*, 2001; Tarayre *et al.*, 2007). Its stems and spines are both photosynthetic and it has few leaves (Clements *et al.*, 2001). It is native to Western Europe and North Africa but during the 20th century its range has expanded and it is now a highly noxious weed in Australia, New Zealand, Chile, Canada, Hawaii, and North America (Clements *et al.*, 2001). *Cassytha pubescens* R. Br. (Lauraceae) is a perennial, coiling hemiparasitic vine 0.5–1.5mm thick that attaches to host stems and leaves via multiple haustoria (McLuckie, 1924; Weber, 1981). It has highly reduced leaves and its stems are photosynthetic (Prider *et al.*, 2009). It is widespread in south-eastern Australia and New Zealand (Weber, 1981) and is frequently found infecting both native and introduced hosts (including *U. europaeus*) in South Australia (Prider *et al.*, 2009; Shen *et al.*, 2010).

### Plant material and growth conditions

*Ulex europaeus* plants, all of around the same size (approximately 30cm tall) and stage of development, were obtained from the field in early July 2013 (Mt. Lofty Ranges, South Australia: S 35º 00.456; E 138º 41.212). Each plant was transplanted into a 1.65 l pot filled with sandy loam. Randomly selected plants were infected with *C. pubescens* using the technique of Shen *et al.* (2010). Briefly, they were placed adjacent to large *U. europaeus* plants already infected with *C. pubescens,* allowing single stems of the parasite to attach to each new host. The connection with the donor host was severed in late November 2013, three months after infection was initiated. Newly attached *C. pubescens* were monitored for a further week to ensure that infection was successful. During the establishment of infection, all *U. europaeus* plants were provided with Nitrosol at rates recommended by the manufacturer (Rural Research Ltd, Auckland, New Zealand; NPK 8:3:6 wt. %). Individual plants, both infected and uninfected, were transplanted into 5.0 l pots in mid-December 2013 with the same sandy loam soil and provided with a single, recommended dose of Osmocote (Scotts-Sierra Horticultural Products, Marysville, OH, USA).

The experiment was carried out in an evaporatively cooled glasshouse at the University of Adelaide. Two watering regimes were established based on the field capacity of the soil which was determined using the filter-papertechnique (Bouyoucos, 1929), but slightly modified as a vacuum was not required in this case. Briefly, 20g of dry soil was made into a slurry using water and then poured into a filter paper and allowed to drain for 1hr. The soil was then re-weighed and the field capacity (FC) calculated using the following formula:

$$\text{FC}=\left({\text{S}}_{w}-S_{\text{D}}\right)/S_{\text{D}}$$

where S_w_ is the mass of the drained soil and S_D_ is the mass of the dry soil. In this case, the FC of the soil was 0.32. Thus, the mass of a 5.0 l pot of soil at 100% FC=1.32 × dry mass of soil in the pot (HW treatment=5.0kg). Field capacity at 55% was 0.55×0.32=0.176. Thus, the mass of the 5.0 l pot at 55% FC was 1.176 × dry mass of soil in the pot (LW treatment=4.5kg). Field capacity of 55% for the LW treatment was chosen because previous experiments in our laboratory (data not shown) had demonstrated that the parasite wilted below 55% while, by comparison, *U. europaeus* wilted at 40% FC. Uninfected and infected plants were randomly allocated into the HW or LW treatments and there were four blocks containing all combinations of treatments. Pots in each treatment were weighed and watered accordingly, daily or every second day on cloudy days and re-randomized within each block fortnightly to negate small light differences in the glasshouse. Watering treatments ran from mid-February to mid-April 2014 when the plants were harvested.

### Host and parasite chlorophyll a fluorescence

Photosynthetic light-use efficiency of *U. europaeus* and *C. pubescens* was measured using a portable, pulse-modulated chlorophyll fluorometer (Mini-PAM, Walz, Effeltrich, Germany) equipped with a leaf-clip (2030–B, Walz, Effeltrich, Germany). Pre-dawn (*F*_v_/*F*_m_) and midday (Φ_PSII_) quantum yields (Genty *et al.*, 1989) were measured on *U. europaeus* spines, and also 15cm from the growing tip of parasite stems 46 days after treatments had been imposed (DAT). Midday measurements were made on a sunny day between 12–1 pm at a photosynthetic photon flux density (PPFD) of approximately 1200 μmol m^–2^ s^–1^.

### Host water potentials

Midday shoot water potentials (Ψ) of *U. europaeus* were measured on freshly cut shoots using a Scholander-type pressure chamber with a digital gauge (PMS Instrument Company, Albany, OR). The balancing pressure was recorded once xylem sap had first appeared. Measurements were made between 1–2 pm (daylight saving time) on a sunny day 52 DAT. Water potential measurements on the parasite were not possible due to insufficient quantities of parasite tissue and also because the morphology of the parasite makes it very difficult to obtain Ψ measurements using a pressure chamber.

### Host and parasite biomass, δ^13^C, nitrogen, and sodium concentration

The shedding of plant tissue in response to infection did not take place during the experiment (personal observations). Unfortunately, an initial harvest to enable quantification of host/parasite growth increments over the experimental period was not possible because of pre-experimental plant mortality leaving *n*=4. A final harvest was conducted 60 DAT with plants divided into spines (no leaves present), stems, roots, and nodules, and separated from parasite stems in the case of infected hosts. Both host and parasite material was oven-dried at 60 ºC for 6 d. The spine area was calculated using previously determined positive linear relationships between spine weight and area for each treatment combination (all *R* >0.99) (Rolston and Robertson, 1976).

Stable carbon isotope composition and nitrogen concentration of host spines and parasite stems were determined using a Horizon isotope ratio mass spectrometer (Nu Instruments Ltd., Wrexham, UK) and a Euro elemental analyser (EuroVector, Tortona, Mil.) at the University of Adelaide. Sodium content of host spines and parasite stems was quantified with the Spectro CIROS CCD Radial Inductively Coupled Plasma Optical Emission Spectrometer (SPECTRO Analytical Instruments GmbH, Kleve, Germany) at Waite Analytical Services (University of Adelaide). All analyses were conducted on final harvest oven-dried material.

### Statistical analysis

The variances of the data were homogenous and a two-way ANOVA was used to test for infection and water effects on *U. europaeus*. The additive effects of infection; comparisons between uninfected (uninfected HW and LW plants pooled) and infected (infected HW and LW plants pooled) plants, or the additive effects of water; comparisons between HW (uninfected and infected HW plants pooled) and LW (uninfected and infected LW plants pooled) plants were only considered if the interaction between infection×water was not significant. One-way ANOVA was conducted on *C. pubescens* data to test for any effects of water. Interactions and additive significant effects of infection or water generated by a Standard least squares model were only considered when pairwise comparisons of means were significant using a Tukey–Kramer HSD test. All data were analysed with the software JMP Ver. 4.0.3 (SAS Institute Inc., 2000) and *α*=0.05.

## Results

### Quantum yields of host and parasite

**Fig. 1.**
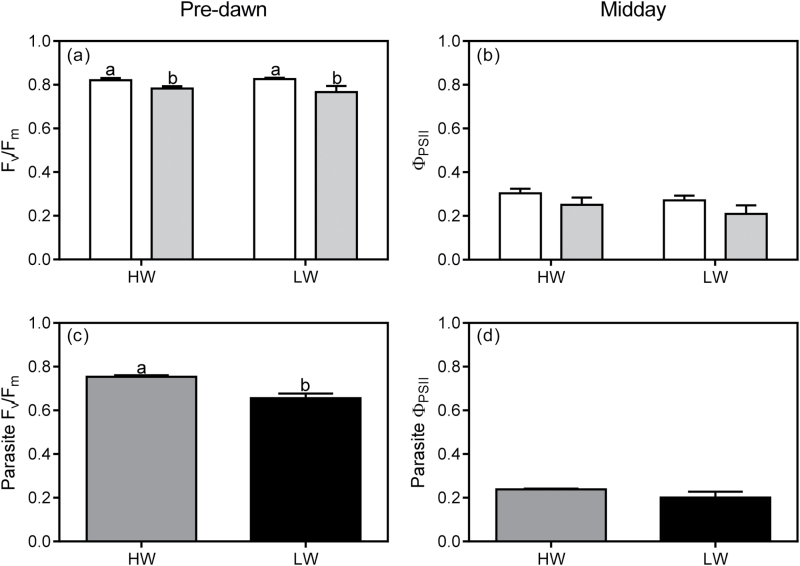
(a) Pre-dawn (*F*_v_/*F*_m_) and (b) midday (Φ_PSII_) quantum yields of *U. europaeus* uninfected (open bars) or infected (grey bars) with *C. pubescens* in high (HW) or low (LW) water conditions. (c) *F*_v_/*F*_m_ and (d) Φ_PSII_ of *C. pubescens* infecting *U. europaeus* in HW (dark grey bars) or LW (black bars) conditions. Different letters denote significant differences, data are means (±1 SE) and *n*=4.

There was no interaction between infection × water for *F*_v_/*F*_m_ or Φ_PSII_ of *U. europaeus* (Table 1; Fig. 1a, b). There was, however, an independent effect of infection on *F*_v_/*F*_m_ but not on Φ_PSII_ (Table 1; Fig. 1a). On average, *F*_v_/*F*_m_ of infected plants (0.775±0.014) was 6% lower than that of uninfected plants (0.823±0.006), regardless of watering treatment. There were no significant independent effects of watering on host *F*_v_/*F*_m_ or Φ_PSII_ (Table 1).

**Table 1.** Results of two-way ANOVA on the additive effects of infection with *C. pubescens* (I), watering treatment (W), and their interaction I×W on pre-dawn and midday quantum yields (*F*_v_/*F*_m_, Φ_PSII_) of *U. europaeus* **P**, *F*, and sum of square values are in bold, italic, and regular type, respectively, and *df*=1, 9 for all parameters.

	*F*_v_/*F*_m_	Φ_PSII_
I	**0.019**	**0.121**
	*8.14*	*2.94*
	0.009	0.013
W	**0.743**	**0.299**
	*0.114*	*1.21*
	0.0001	0.005
I×W	**0.525**	**0.893**
	*0.438*	*0.019*
	0.0005	0.00009
Block	**0.663**	**0.896**
	*0.546*	*0.196*
	0.002	0.003
Error	0.010	0.040

*F*_v_/*F*_m_ of *C. pubescens* was significantly affected by water (Table 2). *F*_v_/*F*_m_ of the parasite in LW was 13% lower relative to that in HW conditions (Fig. 1c). There was no effect of water on parasite Φ_PSII_ when measured under prevailing light conditions at midday (Table 2; Fig. 1d).

**Table 2.** Results of one-way ANOVA on effects of watering treatment (W) on pre-dawn and midday quantum yields (*F*_v_/*F*_m_, Φ_PSII_), carbon isotope composition (δ^13^C), stem nitrogen (N) and sodium (Na) concentration, parasite biomass, and parasite biomass g^−1^ host biomass of *C. pubescens* when infecting *U. europaeus* **P**, *F*, and sum of square values are in bold, italic, and regular type, respectively, and *df*=1, 3 for all parameters.

	*F*_v_/*F*_m_	Φ_PSII_	δ^13^C	N	Na	Biomass	Biomass g^−1^ host biomass
W	**0.011**	**0.265**	**0.001**	**0.426**	**0.011**	**0.118**	**0.069**
	*33.0*	*1.87*	*135*	*0.843*	*32.7*	*4.71*	*7.78*
	0.019	0.003	4.62	0.061	94531250	59.8	0.382
Block	**0.264**	**0.550**	**0.155**	**0.337**	**0.465**	**0.333**	**0.297**
	*2.23*	*0.853*	*3.72*	*1.70*	*1.12*	*1.73*	*1.96*
	0.004	0.004	0.381	0.370	9693750	65.7	0.289
Error	0.002	0.005	0.103	0.218	8673750	38.1	0.147

### Host and parasite biomass

**Fig. 2.**
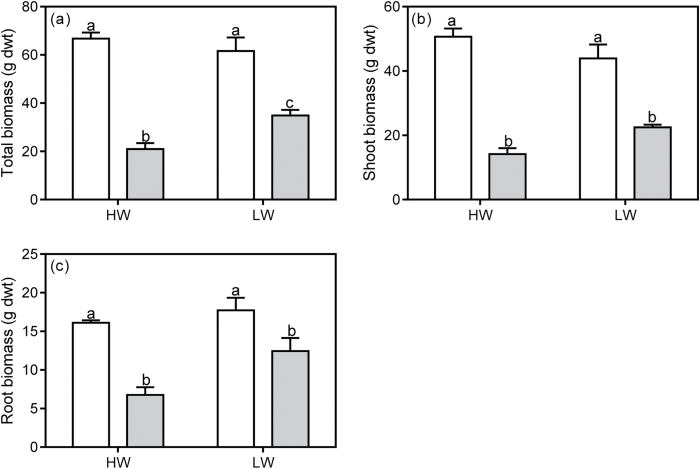
(a) Total, (b) shoot, and (c) root biomass (g dwt) of *U. europaeus* either uninfected (open bars) or infected (grey bars) with *C. pubescens* in high (HW) or low (LW) water conditions. Different letters denote significant differences, data are means (±1 SE) and *n*=4.

**Fig. 3.**
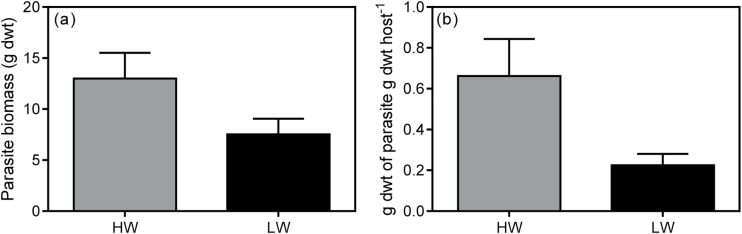
(a) Parasite biomass (g dwt) and (b) parasite biomass supported per unit host biomass (g dwt g^–1^ dwt host biomass) of *C. pubescens* infecting *U. europaeus* in high (HW, dark grey bars) or low (LW, black bars) water conditions. No significant differences were detected, data are means (±1 SE) and *n*=4.

Infection had a differential impact on total biomass of *U. europaeus* in HW and LW (significant interaction, Table 3; Fig. 2a). Infection decreased total biomass of *U. europaeus* by 69% and 43% in the HW and LW treatments, respectively (Fig. 2a). Although there was a significant interaction for shoot biomass which followed a similar pattern, no significant difference was detected by the pairwise comparison (Table 3; Fig. 2b). Root biomass also followed a similar trend but no interaction was detected (Table 3; Fig. 2c). However, there were significant infection effects on both shoot and root biomass (g dwt) (Table 3; Fig. 2b, c). On average, shoot biomass of infected plants (18.3±1.8) was approximately 60% lower compared with that of uninfected plants (47.3±2.6), irrespective of watering treatment. In addition, root biomass of infected *U. europaeus* (9.6±1.4) was 43% lower than that of uninfected plants (16.9±0.8). There was a trend for the biomass of *C. pubescens* to be higher on HW than LW hosts and this difference was marginally significant on a per unit host biomass basis (*P*=0.069) (Table 2; Fig. 3a, b).

**Table 3.** Results of two-way ANOVA on the additive effects of infection with *C. pubescens* (I), watering treatment (W), and their interaction I×W on total, shoot, and root biomass, spine area (SA), shoot/root ratio (S/R), nodule biomass (Nod), and Nod g^−1^ root biomass of *U. europaeus* **P**, *F*, and sum of square values are in bold, italic, and regular type, respectively, and *df*=1, 9 for all parameters. Although the interaction for shoot biomass was significant, because the pairwise comparison did not detect these differences this effect was not considered.

	Total	Shoot	Root	SA	S/R	Nod	Nod g^−1^ root
I	**<0.0001**	**<0.0001**	**<0.0001**	**<0.0001**	**0.005**	**0.0008**	**0.0006**
	*186*	*178*	*45.8*	*226*	*13.5*	*24.5*	*26.4*
	5263	3355	214	765822	2.46	0.295	0.0008
W	**0.132**	**0.733**	**0.008**	**0.049**	**0.051**	**0.035**	**0.032**
	*2.74*	*0.124*	*11.4*	*5.18*	*5.08*	*6.16*	*6.38*
	77.7	2.34	53.1	17508	0.922	0.074	0.0002
I×W	**0.006**	**0.007**	**0.092**	**0.003**	**0.429**	**0.081**	**0.075**
	*12.9*	*12.0*	*3.56*	*16.8*	*0.686*	*3.87*	*4.07*
	365	226	16.6	56658	0.125	0.047	0.0001
Block	**0.048**	**0.078**	**0.114**	**0.051**	**0.313**	**0.747**	**0.423**
	*3.95*	*3.17*	*2.63*	*3.82*	*1.37*	*0.415*	*1.03*
	336	179	36.8	38780	0.746	0.015	0.00009
Error	255	170	42.0	30448	1.63	0.109	0.0003

The spine area (SA) of *U. europaeus* was affected in a non-independent way by infection and water (significant interaction; Table 3). Infection decreased spine area by 83% and 51% in the HW and LW treatments, respectively (Table 4). There was no interaction detected for shoot/root ratio, nodule biomass or nodule biomass g^–1^ root biomass, and these parameters were affected only by infection (Table 3). The shoot/root ratio of infected plants was 28% lower compared with that of uninfected plants (Table 4). Nodule biomass of infected plants was an order of magnitude lower relative to that of uninfected plants, and infection decreased nodule biomass g^–1^ root biomass by 82% (Table 4).

**Table 4.** Spine area (SA, cm^2^), shoot/root ratio (S/R), nodule biomass (Nod, g dwt), Nod g^−1^ root biomass, water potential (Ψ, MPa), and carbon isotope values (δ^13^C, ‰) of *U. europaeus*, either uninfected (–) or infected (+) with *C. pubescens* under high (HW) or low (LW) water supply Data are means (±1 SE) and letters denote significant differences for interaction between infection (I) × water (W) for SA (*n*=4), additive (I) effect for S/R, Nod, and Nod g^−1^ root, and additive (W) effect for Ψ (*n*=8). Additively, although the effect of (I) on δ^13^C and (W) on S/R, Nod, Nod g^−1^ root, and δ^13^C was significant, it was not considered because the pairwise comparison did not detect any difference.

	SA	S/R	Nod	Nod g^−1^ root	Ψ	δ^13^C
HW-	672.0±31.7a	3.15±0.170	0.180±0.073	0.011±0.004	−1.91±0.075	−29.2±0.372
LW-	619.1±63.2a	2.49±0.184	0.424±0.069	0.024±0.003	−2.67±0.006	−28.2±0.280
HW+	115.4±17.8b	2.19±0.310	0.016±0.009	0.003±0.002	−1.98±0.043	−29.7±0.627
LW+	300.6±21.3c	1.89±0.199	0.045±0.012	0.004±0.002	−2.76±0.221	−29.5±0.304
Infection						
–	–	2.82±0.170a	0.302±0.066a	0.017±0.003a	−2.29±0.148	−28.7±0.290
+	–	2.04±0.180b	0.030±0.009b	0.003±0.001b	−2.44±0.199	−29.6±0.326
Water						
HW	–	2.67±0.244	0.098±0.046	0.007±0.003	−1.95±0.042a	−29.5±0.350
LW	–	2.19±0.170	0.234±0.079	0.014±0.004	−2.71±0.086b	−28.9±0.309

### Ψ, δ^13^C, and tissue N and Na concentrations

There was no interaction between infection × water or independent infection effect for Ψ of *U. europaeus*, but this parameter was affected by water treatment (Table 5). Water potentials of *U. europaeus* under LW were 28% lower than those of HW plants (Table 4). There was no significant interactive effect on δ^13^C values of *U. europaeus* and, although the model detected a significant additive infection effect, the Tukey test did not find a difference (Tables 4, 5). There was a significant effect of water on δ^13^C of *C. pubescens* (Table 2). Parasite δ^13^C in LW (−26.7±0.149‰) was 5% higher compared with that in HW conditions (−28.2±0.135‰) (significant water effect; Table 2). Also, the carbon isotope composition of *C. pubescens* was significantly higher (species effect, *P* <0.0001) than that of the uninfected and infected hosts in both water treatments (Table 4) (no species × water interaction).

**Table 5.** Results of two-way ANOVA on the additive effects of infection with *C. pubescens* (I), watering treatment (W), and their interaction I×W on water potential (Ψ), carbon isotope values (δ^13^C), spine nitrogen and sodium concentrations of *U. europaeus* **P**, *F*, and sum of square values are in bold, italic, and regular type, respectively, and *df*=1, 9 for all parameters.

	Ψ	δ^13^C	N	Na
I	**0.245**	**0.044**	**0.044**	**0.116**
	*1.55*	*5.51*	*5.51*	*3.02*
	0.092	3.13	0.286	40322500
W	**<0.0001**	**0.129**	**0.221**	**0.058**
	*47.4*	*2.79*	*1.73*	*4.73*
	2.80	1.59	0.090	63202500
I×W	**0.546**	**0.322**	**0.865**	**0.032**
	*0.394*	*1.10*	*0.031*	*6.47*
	0.023	0.624	0.002	86490000
Block	**0.722**	**0.193**	**0.639**	**0.900**
	*0.453*	*1.94*	*0.586*	*0.191*
	0.080	3.31	0.091	7660000
Error	0.532	5.12	0.467	120245000

**Fig. 4.**
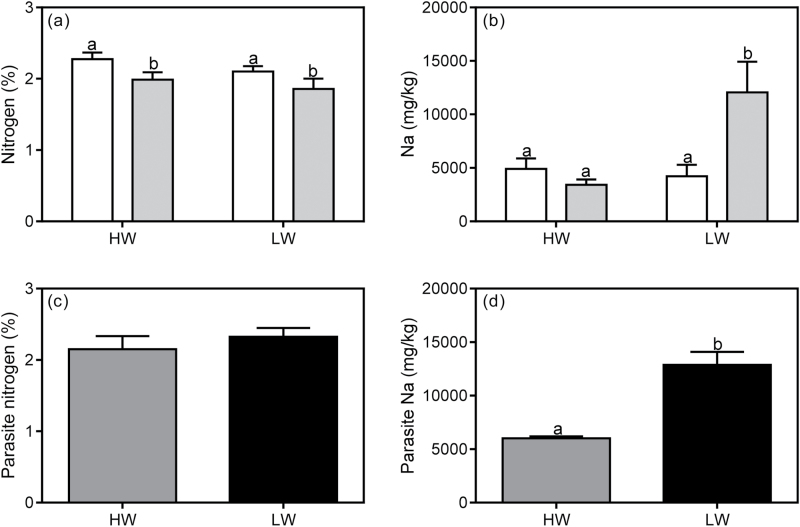
(a) Spine nitrogen (% dwt) and (b) sodium (mg kg^−1^) concentration of *U. europaeus* either uninfected (open bars) or infected (grey bars) with *C. pubescens* in high (HW) or low (LW) water conditions. (c) Stem nitrogen and (d) sodium concentration of *C. pubescens* infecting *U. europaeus* in HW (dark grey bars) or LW (black bars) conditions. Different letters denote significant differences, data are means (±1 SE) and *n*=4.

There was no interactive effect of infection × water for spine nitrogen concentration of *U. europaeus*, but it was affected by infection (Table 5; Fig. 4a). On average, nitrogen concentration (%) of infected plants (1.92±0.09) was 12% lower than that of uninfected plants (2.19±0.06). By contrast, there was a significant interaction between infection × water on the sodium concentration of *U. europaeus* spines (Table 5). There was no effect of the parasite in HW conditions, whereas in LW, the sodium concentration increased by 65% in response to infection (Fig. 4b).

Water had no effect on the stem nitrogen concentration of *C. pubescens* (Table 2; Fig. 4c). By contrast, there was an effect of water on the sodium concentration of *C. pubescens* (Table 2). The sodium concentration of the parasite in LW was 2-fold higher relative to that in HW conditions (Fig. 4d).

## Discussion

The hypothesis that *C. pubescens* would have a negative effect on *U. europaeus*, and that it would be more severe in the HW treatment was supported by the results presented here. Indeed, infection decreased total biomass of *U. europaeus* by nearly 30% more when plants were in HW compared with LW conditions. Similarly, Evans and Borowicz (2013) found that shoot and root biomass of *Verbesina alternifolia* were affected by the stem holoparasitic vine *Cuscuta gronovii*, and these effects were stronger in well-watered relative to dry conditions. Our finding may be due to hosts with a much higher water status (additive water effect; Table 2) possibly permitting higher transpiration rates in the parasite and thus greater resource uptake. This would lead to greater parasite growth and, in turn, further removal of resources from the host that could otherwise be used for photosynthesis and growth.

Following on, *C. pubescens* had higher biomass per unit of host biomass in HW compared with LW conditions, although this was only significant at α <0.07. Similarly, *Cuscuta gronovii* grew significantly larger in absolute and per unit host biomass terms in wet than in droughted treatments (Evans and Borowicz, 2015). As mentioned above, parasite growth in HW may have been greater because of increased resource removal from the host, but also because of increased photosynthesis in the parasite. The decrease in parasite biomass per unit host under LW may be directly due to the relatively high Na concentration in *C. pubescens* in these conditions (Table 2; Figs 3b, 4d) (Taiz and Zeiger, 2002). It may also be due to the much lower *F*_v_/*F*_m_ of the parasite in LW which is evidence of chronic photoinhibition in *C. pubescens,* compared with HW conditions (Demmig-Adams and Adams, 2006). Inoue *et al.* (2013) on the other hand, found no effect of water on *F*_v_/*F*_m_ of *S. hermonthica* infecting sorghum, however, it should be kept in mind that drought treatments in this study only lasted 1–2 d. Here, the relatively high Na concentration in the parasite in LW may also directly explain the decrease in parasite *F*_v_/*F*_m_ and or indirectly given that it may affect gas exchange, e.g. stomatal conductance (James *et al.*, 2002; Taiz and Zeiger, 2002; Parida and Das, 2005; Ranjbarfordoei *et al.*, 2006). The fact that δ^13^C of *C. pubescens* was significantly higher in LW than in HW conditions does infer that the parasite maintained lower stomatal conductances in LW (Scalon and Wright, 2015). This may also have occurred if the parasite found it increasingly difficult to extract water from the hosts under the LW treatment, which could be likely given that host Ψ was significantly lower in these conditions (Table 4). Declines in parasite *F*_v_/*F*_m_ in the LW treatment could also have occurred if stem N concentration was lower, however, this parameter was unaffected by watering treatment (Fig. 4c).

Infection had a negative effect on *F*_v_/*F*_m_ of *U. europaeus*, regardless of water treatment. On the other hand, Le *et al.* (2015) found that a fluorescence parameter used as a proxy for *F*_v_/*F*_m_ of *Mikania micrantha* was negatively affected by *Cuscuta australis* in droughted but not in well-watered treatments. Here, infection effects may, in part, be due to the negative effect of *C. pubescens* on the N concentration of *U. europaeus* (additive infection effect; Table 5; Fig. 4a). A similar explanation was provided for the strong decline in apparent quantum yield of *M. micrantha* in response to infection with *Cuscuta campestris* (Shen *et al.*, 2013). Moreover, depressions in *F*_v_/*F*_m_ of some plant species have resulted from N deficiency (Verhoeven *et al.*, 1997; Huang *et al.*, 2004; Zhou *et al.*, 2006). Ultimately, our finding may be explained by the removal of N by the parasite. Infection negatively affecting host nitrogen would probably affect photosynthetic performance and should result in less carbohydrate which would explain significant infection effects on nodulation and nodulation per unit root biomass which might further limit the acquisition of N by infected plants.

Interestingly, infection had no effect on the Ψ of *U. europaeus*, in either HW or LW conditions. Similarly, Inoue *et al.* (2013) also found no effect of the root hemiparasite *S. hermonthica* on the relative water content of sorghum in either wet or dry treatments. The lack of an infection effect of host Ψ may be due to infected plants having lower stomatal conductances which would ameliorate their water status; but their more negative δ^13^C does not support this notion. A more likely explanation may be related to significant reductions in host growth. All things being equal, a smaller infected plant requires less water than a larger uninfected plant to maintain similar water potentials. Further, although, infected hosts in LW received less water than smaller HW infected hosts, it is likely that the parasite also removed less water in these conditions due to stomatal limitations as inferred from the carbon isotope composition of the parasite mentioned earlier. In addition, infected LW hosts were significantly enriched in sodium (with respect to all other plants) which would make their osmotic potential and thus, water potential more negative. This would have the dual benefit of facilitating water uptake from the soil and impeding water removal by *C. pubescens* in this treatment. Infected LW plants did have the lowest water potentials, which is consistent with this argument.

This experiment clearly demonstrated that the impact of *C. pubescens* on total biomass of *U. europaeus* was more severe under conditions of high water availability. This may be due to a well-hydrated host resulting in a well-hydrated, healthy parasite that is capable of maintaining higher stomatal conductance (δ^13^C) and, hence, removing more resources from the host. Importantly, δ^13^C of the parasite was significantly higher than that of both uninfected and infected *U. europaeus*, suggesting that the parasite was more conservative in its water use than the host. To our knowledge, this finding has not previously been reported for stem hemiparasitic plant–host associations. By contrast, Scalon and Wright (2015), looking at the δ^13^C of 168 mistletoe–host pairs from 39 sites across the globe, in general, found the opposite to be true. This discrepancy between findings may be due to mistletoes mainly infecting trees that would have a much larger root system and hence have access to more water than plants in pots. Nevertheless, Scalon and Wright (2015) showed that mistletoes and their hosts save more water as moisture decreases. Here, the carbon isotope composition of the plants is in line with this, inferring that *C. pubescens* maintained lower stomatal conductances in LW (Scalon and Wright, 2015) and, in this case, even more so than the host. From the above, it was speculated that water supply, in conjunction with size of host roots and surface area of the parasite, may dictate the performance of *C. pubescens*. This was corroborated by the fact that *C. pubescens* was observed to wilt (below 55% FC) well before *U. europaeus* (40% FC) (personal observations).

From the evidence, it is concluded that, when infected with *C. pubescens*, the growth of *U. europaeus* would decrease in mesic conditions more than in drier conditions. Nonetheless, even in times of prolonged drought, which are predicted as a consequence of climate change for many of the regions where *U. europaeus* occurs, the data clearly indicate that *C. pubescens* will still have a strong impact on the biomass of *U. europaeus*.

## Supplementary data

Supplementary data can be found at *JXB* online.

Supplementary Fig. S1. Photos of the stem hemiparasite *Cassytha pubescens* growing on the introduced host *Ulex europaeus* in high (HW) and low (LW) water treatments.

Supplementary Fig. S2. Close-up photos of *C. pubescens* growing tips when infecting *U. europaeus* in HW and LW treatments.

Supplementary Data
