# Supplementary material for: High water availability increases the negative impact of a native hemiparasite on its non-native host
Source: J Exp Bot. 2015 Dec 23;67(5):1567–75. doi: 10.1093/jxb/erv548 (PMC4762389; doi:10.1093/jxb/erv548)
Supplement: Supplementary Data [file supp_67_5_1567__index.html]

High water availability increases the negative impact of a native hemiparasite on its non-native host — High water availability increases the negative impact of a native hemiparasite on its non-native host — Supplementary Data 

# High water availability increases the negative impact of a native hemiparasite on its non-native host

## Supplementary Data

Data files

- Supplementary\_figures\_S1\_S2.pdf - Supplementary Data
