# Supplementary material for: High water availability increases the negative impact of a native hemiparasite on its non-native host
Source: J Exp Bot. 2015 Dec 23;67(5):1567–75. doi: 10.1093/jxb/erv548 (PMC4762389; doi:10.1093/jxb/erv548)
Supplement: Supplementary Data [file supp_erv548_Supplementary_figures_S1_S2.pdf]

Journal of Experimental Botany

High water availability increases the negative impact of a native hemiparasite on its non-native host.

Robert M. Cirocco, José M. Facelli, Jennifer R. Watling

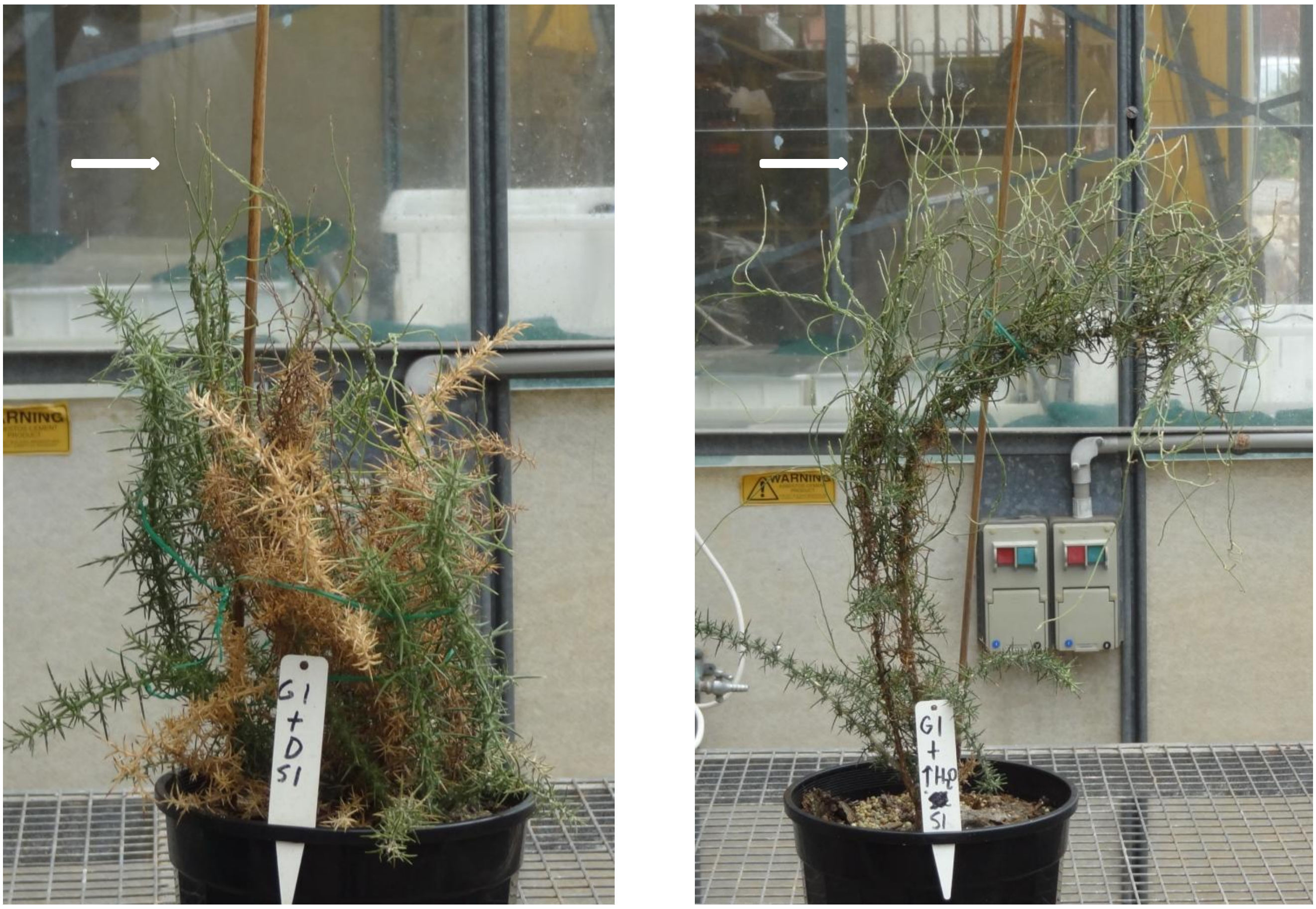

Figure S1. *Ulex europaeus* plants infected with the stem hemiparasite *Cassytha pubescens* (arrow) from the LW (left) or HW (right) treatments.

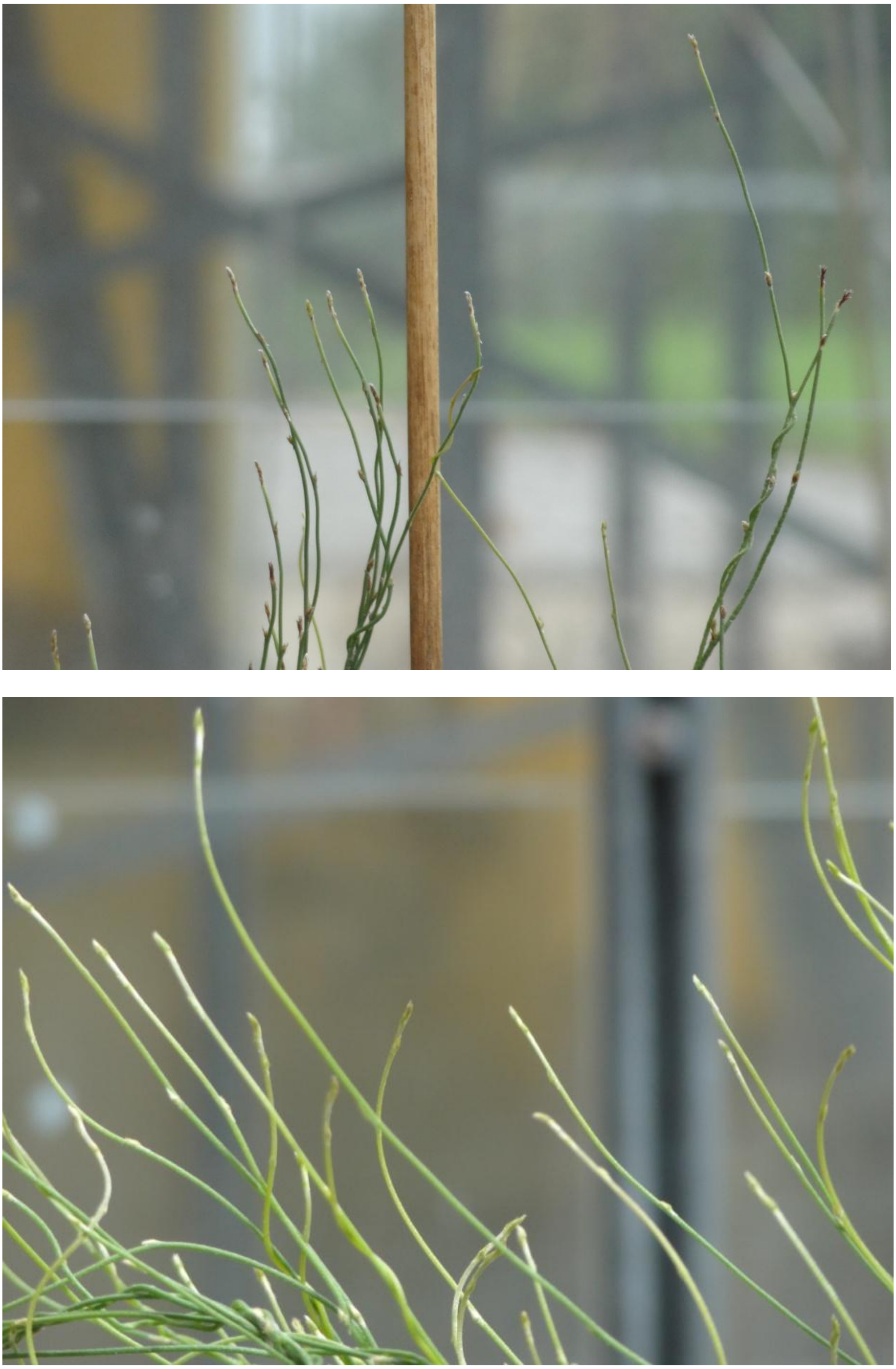

Figure S2. Close up of tips of the stem hemiparasite *C. pubescens* when infecting *U. europaeus* in the LW (top) or HW (bottom) treatments.
